# Development of a standard of care for patients with valosin-containing protein associated multisystem proteinopathy

**DOI:** 10.1186/s13023-022-02172-5

**Published:** 2022-01-29

**Authors:** Manisha Korb, Allison Peck, Lindsay N. Alfano, Kenneth I. Berger, Meredith K. James, Nupur Ghoshal, Elise Healzer, Claire Henchcliffe, Shaida Khan, Pradeep P. A. Mammen, Sujata Patel, Gerald Pfeffer, Stuart H. Ralston, Bhaskar Roy, William W. Seeley, Andrea Swenson, Tahseen Mozaffar, Conrad Weihl, Virginia Kimonis, the VCP Standards of Care Working Group, Roberto Fanganiello, Grace Lee, Ryan Patrick Mahoney, Jordi Diaz-Manera, Teresinha Evangelista, Miriam Freimer, Thomas E. Lloyd, Benison Keung, Hani Kushlaf, Margherita Milone, Merrilee Needham, Johanna Palmio, Tanya Stojkovic, Rocío-Nur Villar-Quiles, Leo H. Wang, Matthew P. Wicklund, Frederick R. Singer, Mallory Jones, Bruce L. Miller, S. Ahmad Sajjadi, Andre Obenaus, Michael D. Geschwind, Ammar Al-Chalabi, James Wymer, Nita Chen, Katie Kompoliti, Stephani C. Wang, Catherine A. Boissoneault, Betsaida Cruz-Coble, Kendrea L. Garand, Anna J. Rinholen, Lauren Tabor-Gray, Jeffrey Rosenfeld, Ming Guo, Nathan Peck

**Affiliations:** 1 Department of Neurology, University of California - Irvine School of Medicine, Orange, CA USA; 2 Department of Pediatrics, University of California - Irvine School of Medicine, Orange, CA USA; 3 Department of Pathology & Laboratory Medicine, University of California - Irvine School of Medicine, Orange, CA USA; 4 Cure VCP Disease, Americus, GA USA; 5 The Abigail Wexner Research Institute at Nationwide Children’s Hospital, Columbus, OH USA; 6 Department of Medicine (Pulmonary), NYU Grossman School of Medicine, New York, NY USA; 7 The John Walton Muscular Dystrophy Research Centre, Newcastle University and Newcastle Hospitals NHS Foundation Trust, Newcastle Upon Tyne, UK; 8 Department of Neurology and Psychiatry, Washington University in St. Louis, St. Louis, MO USA; 9 Thriving Hope Consulting, Vinton, IA USA; 10 Department of Neurology & Neurotherapeutics, University of Texas Southwestern Medical Center, Dallas, TX USA; 11 Department of Medicine (Cardiology), University of Texas Southwestern Medical Center, Dallas, TX USA; 12 Wellness with Sujata, Wadsworth, OH USA; 13 Hotchkiss Brain Institute, University of Calgary Cumming School of Medicine, Calgary, AB Canada; 14 Institute of Genetics and Cancer at the University of Edinburgh, Edinburgh, SCT UK; 15 Department of Neurology, Yale School of Medicine, New Haven, CT USA; 16 Weill Institute for Neurosciences, University of California San Francisco, San Francisco, CA USA; 17 Department of Neurology, University of Iowa Hospitals and Clinics, Iowa City, IA USA; 18 Department of Neurology, Washington University in St. Louis, St. Louis, MO USA; 19 The Hope Center, Washington University in St. Louis, St. Louis, MO USA

## Abstract

Valosin-containing protein (VCP) associated multisystem proteinopathy (MSP) is a rare inherited disorder that may result in multisystem involvement of varying phenotypes including inclusion body myopathy, Paget’s disease of bone (PDB), frontotemporal dementia (FTD), parkinsonism, and amyotrophic lateral sclerosis (ALS), among others. An international multidisciplinary consortium of 40+ experts in neuromuscular disease, dementia, movement disorders, psychology, cardiology, pulmonology, physical therapy, occupational therapy, speech and language pathology, nutrition, genetics, integrative medicine, and endocrinology were convened by the patient advocacy organization, Cure VCP Disease, in December 2020 to develop a standard of care for this heterogeneous and under-diagnosed disease. To achieve this goal, working groups collaborated to generate expert consensus recommendations in 10 key areas: genetic diagnosis, myopathy, FTD, PDB, ALS, Charcot Marie Tooth disease (CMT), parkinsonism, cardiomyopathy, pulmonology, supportive therapies, nutrition and supplements, and mental health. In April 2021, facilitated discussion of each working group’s conclusions with consensus building techniques enabled final agreement on the proposed standard of care for VCP patients. Timely referral to a specialty neuromuscular center is recommended to aid in efficient diagnosis of VCP MSP via single-gene testing in the case of a known familial VCP variant, or multi-gene panel sequencing in undifferentiated cases. Additionally, regular and ongoing multidisciplinary team follow up is essential for proactive screening and management of secondary complications. The goal of our consortium is to raise awareness of VCP MSP, expedite the time to accurate diagnosis, define gaps and inequities in patient care, initiate appropriate pharmacotherapies and supportive therapies for optimal management, and elevate the recommended best practices guidelines for multidisciplinary care internationally.

**Supplementary Information:**

The online version contains supplementary material available at 10.1186/s13023-022-02172-5.

## Introduction

Valosin-containing protein (VCP) associated multisystem proteinopathy (MSP) is a rare and heterogeneous genetic disorder that can cause adult-onset inclusion body myopathy, Paget’s disease of bone (PDB), and frontotemporal dementia (FTD), but other associated manifestations include parkinsonism, amyotrophic lateral sclerosis (ALS), neuropathy, spastic paraplegia, and cardiomyopathy, among others (Table 1). The previously used term, inclusion body myopathy associated with Paget’s disease of bone (and frontotemporal dementia (IBMPFD), is no longer favored given the other phenotypes that may occur. Other genes that are associated with a similarly presenting MSP syndrome include heterogeneous nuclear ribonucleoprotein A2B1 and A1 (*hnRNPA2B1, hnRNPA1*), sequestosome 1 (*SQSTM1*), matrin 3 (*MATR3*), T-cell restricted intracellular antigen 1 (*TIA1*), optineurin (*OPTN*), annexin 11 (*ANXA11*), and profilin 1 (*PFN1I*), many of which share common pathophysiology of disruption of RNA stress granule function or autophagic degradation, and patients presenting with these MSP syndromes may benefit from similar diagnostic and treatment strategies [1, 2].

**Table 1:** Clinical manifestations of VCP MSP

Phenotype	System affected	Clinical features	Frequency in VCP patients
Inclusion body myopathy	Muscle	Axial and proximal weakness progressing distally is most common, although presentations resembling facioscapulohumeral muscular dystrophy, oculopharyngeal muscular dystrophy, and distal myopathy have been described	~ 90%
Paget disease of bone (PDB)	Skeletal	Bone pain, bone deformities, pathological fractures, hearing loss	~ 40%
Frontotemporal dementia (FTD)	Cognitive	Rapidly progressive behavioral impairment, executive dysfunction, language impairment. Often associated with Parkinsonian features such as dystonia, tremor, gait disturbance	~ 30%
Respiratory dysfunction	Pulmonary	Recurrent respiratory infections, weak cough, aspiration, sleep disordered breathing, respiratory failure	40–50%
Amyotrophic lateral sclerosis (ALS)	Upper and lower motor neurons	Multifocal weakness, hyperreflexia and/or areflexia, atrophy, fasciculations, bulbar weakness, respiratory muscle involvement, weight loss	~ 10%
Parkinson disease (PD)	Central nervous system	Hypokinetic movement disorder, autonomic dysfunction, various non-motor features	4%
Alzheimer disease (AD)	Cognitive	Dementia with predominant amnestic and higher order cognitive dysfunction	2% [4]
Spastic paraplegia	Upper motor neurons	Length-dependent weakness, hyperreflexia, spasticity, clonus	Isolated cases
Sensorimotor neuropathy (axonal Charcot Marie Tooth disease (CMT))	Peripheral nerves	Length-dependent weakness, muscle atrophy and sensory loss. Trophic foot changes and distal areflexia	Isolated cases
Cardiomyopathy	Cardiac	Exertional shortness of breath, heart failure	Uncertain. Reported in case series
Dysphagia and dysarthria	Bulbar dysfunction	Impaired swallowing function, reduced speech volume and intelligibility	Uncertain
Urinary and anal incontinence	Genitourinary, gastrointestinal	Urinary incontinence, anal incontinence or dysfunction	Uncertain

On April 9, 2021, over 40 specialists attended a virtual international meeting to establish a consensus on standard of care recommendations for the diagnosis and treatment of VCP MSP. The meeting was organized by the patient advocacy organization, Cure VCP Disease, and multidisciplinary physicians and therapists presented on the clinical features, diagnosis, surveillance, and management of the different aspects of the disease. The topics discussed were genetic diagnosis, myopathy, FTD, PDB, ALS, Charcot Marie Tooth disease CMT, Parkinson’s disease (PD)/parkinsonism, cardiomyopathy, respiratory dysfunction, supportive therapies [including physical and occupational therapy (PT/OT), speech-language pathology (SLP)], mental health, supplements, and nutrition. The goal was to develop a robust consensus-based set of recommendations from our experts based on systematic literature review and clinical experience; the recommendations for each sub-category of the disease are highlighted in Table 2. Due to a lack of evidence in VCP disease, we deferred to guidance and experience from diseases with phenotypic similarities to aspects of VCP, consistent with Level III evidence based on the Canadian Task Force on the Periodic Health Examination’s Level of Evidence [3]. This report will summarize the recommendations from the meeting, with the aim of increasing awareness of VCP MSP, expediting time to accurate diagnosis, and establishing a multidisciplinary standard of care for appropriate pharmacotherapies and supportive therapies.

**Table 2:** Summary of screening and treatment recommendations

Domain	Diagnosis	Treatment and surveillance
Genetics	Consider MSP genetic testing in patients with personal or family history of one or more of the described phenotypes including myopathy, PDB, FTD, ALS, CMT, and/or spastic paraplegia Genetic testing is the gold standard for diagnosis of VCP MSP and other genetic causes of MSP Single-gene testing is recommended for patients who have a known familial mutation Multi-gene panel testing is recommended for undifferentiated patients	Confirm VCP inclusion in multi-gene testing panel Genetic counseling
Myopathy	CK can be normal to mildly elevated NCS/EMG can show mixed neurogenic and myopathic features and may help with diagnosis of concomitant neuropathy or motor neuron disease Muscle MRI T1 weight imaging can show “fat pockets” in proximal and distal lower limb muscles Muscle biopsy may show rimmed vacuoles, fiber size variability, ubiquitin and TDP-43 inclusions. It may be helpful in cases of diagnostic uncertainty for other diseases	No disease modifying therapy Supportive management: PT, OT, SLP, RT, mechanical aids PT guidance on appropriate and safe exercise *Surveillance*: Follow with a multidisciplinary team for standardized strength and functional testing using appropriate COA every 6 months to 1 year, or more frequently if weakness progresses more rapidly
Frontotemporal dementia	Screen for FTD with clinical assessment for behavioral and cognitive impairment, and bedside cognitive tools like MoCA and NPI-Q Formal neuropsychiatric testing if FTD is suspected Brain MRI with atrophy of frontal and/or temporal lobes can support diagnosis FDG PET can show hypometabolism of frontal and/or temporal lobes but is not required for diagnosis	No disease modifying therapy Supportive management: avoiding triggers for high-risk behaviors, caregiver support and education Speech language pathology Anti-depressants and anti-psychotics may have some benefit for the treatment of FTD associated behaviors *Surveillance*: Annual neuro assessment with bedside tools like MoCA and NPI-Q
Paget’s disease of the bone	Most sensitive diagnostic test is a radionuclide bone scan X-ray can help evaluate focal bone pain Serum ALP is less sensitive, but typically elevated in the setting of normal liver function tests Biochemical markers such as PINP, BALP, NTX, and CTX are elevated in active PDB but do not offer any clear advantage over ALP	Bisphosphonates can help bone pain *Surveillance*: Check a baseline bone scan, and then recheck if the patient develops symptoms of bone pain in a new site. Serum ALP can be used for follow up every 6–12 months
ALS	Clinical exam shows upper and lower motor neuron signs NCS/EMG shows a disorder of motor neurons	Multidisciplinary clinic with neurologist, PT, OT, SLP, RT, SW, palliative care No consensus on whether to prescribe ALS drugs (riluzole, edaravone) *Surveillance*: If patients’ weakness becomes more rapid, or they develop UMN signs, bulbar weakness, or respiratory dysfunction, they should be evaluated for ALS clinically and with an EMG. They should be followed every 3 months thereafter
CMT	NCS/EMG shows length-dependent axonal sensorimotor peripheral neuropathy Genetic sequencing confirms diagnosis	PT, OT, ambulatory assistive devices Surgical procedures for foot deformities *Surveillance*: Check a baseline NCS/EMG, and surveillance via annual physical exams
Parkinson’s disease/ parkinsonism	Clinical exam shows bradykinesia, muscle rigidity, rest tremor, and postural instability DaTScan shows nigrostriatal dopamine deficit	Trial of Sinemet, which if successful can either be continued or switched to another symptomatic dopaminergic agent *Surveillance*: Check a baseline MDS-UPDRS, and repeat yearly as needed
Cardiomyopathy	Cardiac MRI is the gold standard, although echocardiogram is also useful for assessing cardiac structure and function	ACE inhibitors or ARBs, beta blockers, MRAs AICD or LVAD if severe *Surveillance*: Check a baseline cardiac MRI and repeat if symptoms arise. If abnormal, repeat every 2 years
Respiratory dysfunction	Serum bicarbonate and PFTs (sitting and supine FVC, MIP, MEP, PCF) show signs of respiratory weakness Sleep study for sleep disordered breathing	Respiratory therapy LVR bag, MI-E, suction device Noninvasive positive pressure ventilation (NIPPV) Up to date vaccinations *Surveillance*: Check baseline serum bicarbonate, PFTs, and sleep study. PFTs should be repeated annually unless patients have more rapid rate of decline

### Genetics

VCP is a ubiquitously expressed and abundant ATPase involved with protein degradation and autophagy [5]. VCP mediates ubiquitin-dependent cellular processes through the ubiquitin–proteasome system (UPS), protein quality control, regulation of autophagy, transcription factor processing, membrane fusion, and cellular signaling [6]. Over 50 heterozygous missense mutations in *VCP*, mapped to chromosome 9p13.3-12, cause autosomal dominant VCP MSP [4, 7, 8].

VCP mutations are the most common in genetically diagnosed families with MSP and are designated MSP1 [1]. Other genes associated with MSP may cause a clinically indistinguishable phenotype. MSP2 and MSP3 are caused by mutations in *HNRNPA2B1* and *HNRNPA1*, respectively [9]. MSP4 is caused by mutations in *SQSTM1* [10]. In addition, mutations in *MATR3* are linked to familial myopathy with ALS and have rarely been associated with FTD [11, 12], and may be considered MSP5 [1]. *TIA1* is associated with Welander distal myopathy, ALS, and FTD [13]. *OPTN* mutations are another cause of familial ALS and ALS-FTD [14], and genetic variation in OPTN is a risk factor for development of PDB [14, 15]. Most recently, mutations in *ANXA11* have been associated with IBM with ALS/FTD and have been proposed to be categorized as MSP6 [2]. Missense mutations in *PFN1* are associated with familial ALS whereas PFN1 haploinsufficiency is associated with early onset and polyostotic PDB [16, 17]. Disease caused by PFN1 mutations has recently been proposed as MSP7 [16, 18].

MSP should be considered in patients and families with one or more of the described phenotypes including myopathy, PDB, FTD, ALS, axonal CMT, and/or spastic paraplegia. Genetic testing is the primary and definitive means for diagnosis of VCP MSP and other genetic causes of MSP. Single-gene testing is recommended for patients who have a known familial mutation, while multi-gene panel testing is recommended for undifferentiated patients. Some multi-gene panels for myopathy, skeletal disorders, dementia, ALS, spastic paraplegia, neuropathy, and PD, include the *VCP* gene. However, older panels may not have included VCP as the phenotypic spectrum has expanded.

The identification of a pathogenic VCP variant confirms the diagnosis in a clinically affected individual or provides a pre-symptomatic diagnosis for an unaffected individual. The identification of a likely pathogenic variant may be considered diagnostic with the caveat that further evidence could result in later reclassification of the variant. If an unaffected individual has a positive result, there is a 90% chance that he or she will develop one or more of the features of VCP MSP by the age of 45 [19]. A variant of unknown significance (VUS) is a change in VCP that has not previously been associated with human disease; insufficient data exists to support whether the variant is benign or pathogenic, and therefore such a result needs further analysis as a possible new pathogenic variant before being considered as not clinically actionable. Detailed information regarding genetic variant interpretation and pathogenicity criteria may be found by referring to American College of Medical Genetics criteria [20].

The cardiac and respiratory complications of VCP MSP (see below) may benefit from early screening and management, and may be considered as part of the decision to pursue pre-symptomatic testing for VCP MSP. However presymptomatic testing may also be pursued to help make decisions regarding career, lifestyle, and reproductive planning [21]. Pre-symptomatic patients and patients with limited aspects of the MSP syndrome should be monitored by a healthcare professional on a regular basis to watch for development of clinical disease.

National guidelines vary, however genetic counseling should precede and follow pre-symptomatic genetic testing because of the significant implications including family planning, psychosocial counseling, and early identification and treatment of the varied aspects of this multifaceted disease. There can be psychological challenges encountered as a consequence of both positive and negative pre-symptomatic testing [22]. Genetic counselling follow-up should be offered for both mutation carriers and non-carriers, and clinicians should have awareness that additional mental health support may be of value in both situations [23]. Clinicians should be aware of challenges in genetic test result interpretation and genetic counseling, including the Genetic Non-Discrimination Act (GINA), and psychosocial considerations for both presymptomatic and symptomatic individuals.

## Myopathy

### Clinical features

Myopathy is the most common feature of VCP MSP affecting 90% of the individuals, with proximal predominant muscle weakness as well as frequent distal muscle involvement [4, 24, 25]. The mean age of onset is in the fifth decade [4, 24]. Patients may develop foot drop, scapular winging, and scapuloperoneal weakness; facial weakness is less common [26]. Patients may become wheelchair-dependent, with loss of ambulation within 13 ± 7 years from symptom onset [26].

### Diagnosis

Genetic testing remains the only definitive way to diagnose VCP myopathy. Other laboratory testing and imaging are generally nonspecific but can be supportive in the diagnosis of myopathy. Creatine kinase (CK) value can be normal or mildly elevated [24]. Nerve conduction studies (NCS) can be normal or in some cases show an axonal motor or sensorimotor neuropathy [27]. Electromyography (EMG) can show mixed neurogenic and myopathic features [27]. Electrodiagnostic studies may be helpful for ruling out other disease mimics and for identifying motor neuron disease. Muscle MRI T1 weight imaging has shown fatty infiltration, or “fat pockets,” in both proximal and distal muscles of the lower limbs, with small areas of fat replacement surrounded by areas of normal muscle [28]. There is not enough data on Short TI inversion recovery (STIR) imaging, although in some isolated muscles it could show increased signal that may reflect inflammation [28].

Muscle biopsy can be done if there is diagnostic uncertainty and/or no genetic mutation is identified in genes associated with myopathies. It generally shows nonspecific changes, with fiber size variability, atrophic and hypertrophic fibers, and type 1 fiber predominance. While rimmed vacuoles are considered a hallmark of VCP myopathy, they are not always present and can be seen in other inherited muscle diseases [29, 30]. Congophilic inclusions, cytoplasmic ubiquitin, and TAR DNA-binding protein 43 (TDP-43) positive inclusions may be present [29].

### Treatment

There is no approved disease modifying therapy for VCP myopathy, and the management is supportive. Several agents targeting the autophagy and VCP inhibition have shown some promise in preclinical studies [31–33]. Patients should be followed by a multidisciplinary clinic at least every 6 months; the components of this clinic should include a neurologist, PT, OT, SLP, respiratory therapy (RT), and social worker (SW). The team should manage the symptoms of limb and bulbar weakness, respiratory dysfunction, and muscle cramps. Other supportive measures, such as mechanical aids and weight control to avoid obesity can be beneficial, and these are described in more detail in the Supportive Therapies section of this article. For patients with imminent respiratory and cardiac failure, palliative care and end of life discussions should be initiated. Because VCP-specific data on treatment is lacking, we base this on the accepted approach to other vacuolar myopathies.

### Surveillance

Patients with VCP mutations who do not currently have symptoms of myopathy, and family members who are at risk, should be screened for signs of muscle weakness via standardized strength and functional testing by a multidisciplinary team. If weakness exists, they should be followed clinically for signs of progression and possible intervention every 6 months to annually, or more frequently if progression is more rapid.

## Frontotemporal dementia

### Clinical features

FTD affects about 30% of patients with VCP mutations [4] and typically presents as the behavioral variant FTD (bvFTD) subtype [26], although other FTD syndromes such as forms of primary progressive aphasia (PPA) have been reported [34]. Manifestations depend on the clinical syndrome but may include social-emotional changes (apathy, disinhibition, loss of empathy, compulsive behaviors, and overeating) or language deficits (word-finding difficulties, loss of word comprehension, diminished fluency). The average age of onset of FTD in VCP patients is 55 years, which is earlier than in the general population [4].

### Diagnosis

Diagnosis of FTD starts with a clinical history of typical behavioral impairment and/or language decline that results in a reduced level of functioning. In the presence of such a history, bedside tests like the Montreal Cognitive Assessment (MoCA) can be used to approximate the level of cognitive impairment [35], but such tests do not provide sensitive screening tests for FTD. The Neuropsychiatric Inventory Questionnaire (NPI-Q) is a structured, informant-based tool to ascertain some behavioral symptoms relevant to FTD. The MoCA and NPI-Q are preferred in general practice over more specialized, less accessible FTD screening tools, as the latter require training and experience; no screening tool has been validated for VCP-related FTD at this time. A concerning clinical history, with or without supportive evidence from brief cognitive/behavioral assessments, should prompt a brain MRI, screening dementia laboratory tests, and referral to a memory disorders clinic and/or neuropsychiatric testing, which can provide comprehensive assessment of behavioral and cognitive functioning. MRI is useful to rule out non-degenerative causes and may show frontal, insular, and/or anterior temporal lobe atrophy [36, 37].

Additional tests sometimes used in the assessment of patients with FTD include fluorodeoxyglucose (FDG)-PET or amyloid-PET to rule out Alzheimer disease (AD), and lumbar puncture to rule out FTD mimics such as inflammatory or infectious diseases, or to rule out AD using CSF biomarkers for amyloid beta and phospho-tau [38].

### Treatment

There is no disease modifying treatment for FTD, and we must draw on the standard of care for FTD in general as data on VCP-specific FTD treatment is lacking. Supportive measures such as caregiver education, re-direction techniques, removing triggers for high-risk behaviors, and establishing a daily routine can help improve a patient’s quality of life. Caregiver support is essential, as caregivers are at high risk for burnout. Exercise and cognitive activity may have a protective effect, as an active lifestyle has been associated with slower functional decline in some forms of autosomal dominant FTD [39], although this has not been specifically tested in VCP FTD. Speech therapy may help improve communication skills via Augmentative and Alternative Communication (AAC), with a goal of delaying language decline [40].

Antidepressants such as selective serotonin reuptake inhibitors may help compulsive and overeating symptoms that can occur with FTD [41, 42]. Antipsychotics, such as quetiapine or risperdone, or mood stabilizers such as valproate, may be indicated for disruptive aggressive or agitated behaviors, but these medications, which have serious side effects, should be prescribed under the direction of an expert familiar with pharmacological management of FTD. Drugs approved for the treatment of AD, including rivastigmine, galantamine, donepezil, memantine, and the newly approved drug aducanumab, have either not been studied in FTD or have been proven ineffective [43, 44].

### Surveillance

Symptomatic patients with *VCP* mutations who lack FTD should undergo annual screening with a neurological assessment, informant interview, and bedside assessment tools like MoCA and NPI-Q. Among patients with a known mutation but no symptoms of any kind, surveillance may be calibrated to the patient’s age and the estimated age-of-onset, based on affected members of the family.

## Paget’s disease of the bone

### Clinical features

PDB occurs in roughly 40% of patients with VCP MSP [4] and is characterized by focal abnormalities of bone remodeling due to an increase in osteoclastic bone resorption along with increased and disorganized bone formation. Affected bones can become enlarged and deformed, and this may result in bone pain, pathological fractures, secondary osteoarthritis, deafness, and nerve compression [45]. Some PDB patients may be asymptomatic, however, and disease may be found incidentally through lab or imaging studies [46].

### Diagnosis

When a patient exhibits signs of PDB, the most sensitive diagnostic test is a radionuclide bone scan, which shows intense tracer uptake in affected bones. The bone scan should then be followed by X-ray of sites showing abnormal tracer uptake; X-ray can show cortical thickening, bone expansion, and bone deformity [47]. X-rays can help in evaluating the cause of focal bone pain and in this regard, physicians should remain cognizant of pain resulting from sources other than Paget’s such as osteoarthritis.

In metabolically active PDB, serum alkaline phosphatase (ALP) is typically elevated in the setting of normal liver function tests. However, a normal ALP does not exclude a diagnosis of PDB, especially in early disease. Biochemical markers that reflect increased bone turnover such as procollagen type I intact N-terminal propeptide (PINP), bone-specific alkaline phosphatase (BALP), crosslinked N-telopeptide of type I collagen (NTX), and collagen type 1 C-telopeptide (CTX) are elevated in active PDB but do not offer any clear advantage over ALP except in patients with chronic liver disease where liver enzymes may be elevated. Serum calcium, phosphate urea, and parathyroid hormone (PTH) are usually normal in PDB.

### Treatment

Drawing on the standard of care for PDB in general rather than VCP-specific PDB given lack of available data, symptomatic PDB patients can be referred to a bone specialist—either an endocrinologist or rheumatologist. Bisphosphonates including zoledronic acid, pamidronate, risedronate and alendronic acid are first line treatments, and can help symptoms of bone pain [48]. Treatment courses can be repeated as needed if symptoms recur along with raised ALP or increased uptake on bone scan. Vitamin D deficiency should be corrected prior to administration of bisphosphonates, to mitigate against hypocalcemia [49]. If bisphosphonates are contraindicated, calcitonin may be considered for bone pain, but long-term use has been associated with an increased risk of certain cancers. It is unclear whether early intervention with bisphosphonates in VCP MSP who do not have PDB symptoms would be beneficial. The issue of whether prophylactic therapy is beneficial in early PDB is being investigated in the ZIPP study [ISRCTN11616770] where SQSTM1 mutation carriers (a gene implicated in MSP4) have been treated with zoledronic acid or placebo. This study is expected to report during 2022 [50].

Patients who have concomitant arthritis or nerve compression may require analgesic, neuropathic pain medication, or NSAID therapies. Orthopedic surgery can be required in up to 10% of PDB patients, most commonly for fracture repair, hip or knee arthroplasty, or spinal surgery for spinal stenosis [51].

### Surveillance

For newly diagnosed VCP patients, physicians should check a baseline bone scan to assess disease extent. If the patient develops symptoms of bone pain at a site which was initially unaffected, the scan may be repeated. Serum ALP can be repeated every 6–12 months to assess metabolic activity. If ALP values are raised and accompanied by pain at an affected site this would be an indication for further testing and treatment.

## Amyotrophic lateral sclerosis

### Clinical features

ALS is reported to occur in ~ 10% of VCP patients [4], and is clinically indistinguishable from ALS resulting from other familial or sporadic causes [1]. It is a progressive neurodegenerative disorder that affects both upper and lower motor neurons (UMN, LMN), resulting in muscle atrophy, weakness, fasciculations, and brisk reflexes. Bulbar involvement is characterized by dysphagia and dysarthria. Death typically occurs as a result of respiratory failure.

### Diagnosis

The diagnosis of ALS is based on diagnostic criteria, which has evolved over time [52–54]. Patients present with progressive weakness in bulbar, cervical, thoracic, or lumbar regions, and clinical findings of upper and lower motor neuron involvement [52]. EMG can be a supportive tool for confirming lower motor neuron dysfunction and assessing extent and severity of the disease. It is also helpful in diagnosis of cases of VCP myopathy that appear to be evolving into ALS, and when additional pathology like a compressive neuropathy needs to be evaluated. Serum CK may be normal or mildly increased, but typically does not exceed 1000 U/L. VCP gene testing should be included in familial ALS gene panels any time genetic testing is being performed in ALS, since VCP mutations can be found in sporadic ALS patients.

### Treatment

The mainstay of management of VCP patients with an ALS phenotype is regular follow up in a specialized multidisciplinary clinic on average every 3 months, as is done for ALS patients without VCP mutations. The components of this clinic include a neurologist, PT, OT, SLP, RT, and SW. The team manages the symptoms of limb and bulbar weakness, sialorrhea, respiratory dysfunction, muscle cramps, spasticity, pseudobulbar affect, depression, and anxiety. Interventions include percutaneous endoscopic gastrostomy (PEG) placement, non-invasive positive-pressure ventilation (NIPPV), and rarely, tracheostomy with invasive mechanical ventilation. End-of-life decisions, hospice, and palliative care referrals are discussed with the multidisciplinary care team [55].

Although there is no cure for ALS, two medications, riluzole and edaravone, are FDA-approved for slowing disease progression. The decision to offer these drugs is based on provider and patient preference, as there is no evidence to support benefits or risks in VCP-specific ALS.

### Surveillance

VCP patients should be assessed by a neurologist every 6 months for signs of progression of weakness. If rate of decline becomes more rapid and/or patients develop bulbar or respiratory dysfunction or UMN signs, ALS should be considered. An EMG can support a diagnosis of ALS. At this point, patients should be followed every 3 months in the multidisciplinary clinic.

## Charcot Marie Tooth disease

### Clinical features

CMT, also called hereditary motor and sensory neuropathy (HMSN), refers to familial neuropathies with extensive clinical and genetic heterogeneity. VCP MSP has been associated with autosomal dominant CMT (referred to as CMT2), with the designation CMT2Y (OMIM entry 616687), although reports of these cases remain rare [56, 57]. Outside of cases of CMT2Y, it is unknown if peripheral neuropathy is a coexisting feature of VCP MSP; it may, at times, be secondary to a separate condition such as diabetes.

CMT patients present with progressive length-dependent axonal loss in sensory and motor nerves, with predominant motor features. Weakness and atrophy occur in intrinsic foot muscles causing hammer toes and pes cavus, and the calves and intrinsic hand muscles can be involved as the disease progresses.

### Diagnosis

CMT2Y is not clinically distinguishable from other forms of CMT2, so diagnosis must be achieved through genetic sequencing. An NCS/EMG shows axonal length-dependent sensorimotor neuropathy.

### Treatment

VCP patients with a CMT2Y phenotype should receive supportive care with PT, OT, ambulatory assistive devices such as walking aids, ankle–foot orthotics (AFO) and hand splinting if necessary. Surgical procedures for foot deformities may be considered in some cases as well.

### Surveillance

VCP patients should undergo baseline NCS/EMG at the time of diagnosis in the presence of suspicious clinical features to determine whether an axonal neuropathy is present, but surveillance testing is unlikely to be useful unless patients develop new symptoms of polyneuropathy or compressive neuropathy.

## Parkinson’s disease/parkinsonism

### Clinical features

Four percent of VCP patients have parkinsonism [4], and some patients are diagnosed with PD. Intrafamilial variability in phenotype has been well described for VCP patients and in some includes PD/parkinsonism [58]. Additionally, a link is supported by the finding of a greater prevalence of PD/parkinsonism in those with VCP mutations [4].

Clinical manifestations of PD and parkinsonism include bradykinesia, muscle rigidity, rest tremor, and postural instability [59]. Other motor features may include hypophonia, hypomimia, slowed and uncoordinated movement, stiffness and cramps, dystonia, imbalance, and dysphagia. Non-motor features may include hyposmia, mood disorders and anxiety, rapid eye movement (REM) sleep behavior disorders, autonomic dysfunction, hallucinations, mild cognitive impairment, and dementia.

### Diagnosis

PD and parkinsonism are diagnosed based upon neurological history and exam. Neuroimaging biomarkers reflecting nigrostriatal dopamine deficit include 123I-ioflupane SPECT (DaTscan) or other dopamine transporter radioligands detected by SPECT or PET, and FDOPA-PET. Genetic testing should be considered in PD if young onset, atypical features, or family history, and the presence of other manifestations of VCP MCP such as Paget’s disease, ALS, or other should prompt VCP testing.

### Treatment

Given the lack of data on VCP-specific PD treatment, we recommend standard of care PD treatment for these patients. Treatment response to carbidopa-levodopa for parkinsonism remains the gold standard, although multiple other medications may be advised based upon specific symptoms. If this medication improves symptoms, it can either be continued or switched to alternative symptomatic agents. There is no known case of DBS or other surgical treatment for such patients with VCP mutations to our knowledge in the literature.

### Surveillance

In newly diagnosed VCP patients without significant signs of parkinsonism, it is reasonable to perform regular screening using clinical batteries in a multidisciplinary clinic, possibly yearly. The most important clinical battery tool is the Movement Disorder Society—Unified Parkinson’s Disease Rating Scale (MDS-UPDRS), but other non-motor symptoms and signs are also informative. A remaining major challenge is how to discern whether such rating scales are affected by other manifestations of VCP MSP, such as myopathy.

## Cardiomyopathy

### Clinical features

The prevalence of cardiomyopathy among patients with VCP MSP is unknown but appears to be rare [60, 61]. VCP patients without extensive cardiac involvement may be asymptomatic, but as cardiomyopathy progresses to systolic dysfunction with increasing left ventricular dilation and pressure overload, they are more likely to become symptomatic. Patients with left ventricular failure may develop dyspnea with exercise intolerance. Physical exam findings may reveal signs of a volume overloaded state including elevated jugular venous pressure, hepatic congestion, pleural effusion with crackles, and lower extremity edema. Respiratory and cardiac failure can lead to death between the ages of 40s to 60s [62]. A recent study showed a higher prevalence of diastolic dysfunction in VCP patients indicating it may be an early sign of cardiac involvement [63]. It is important to exclude other etiologies of cardiomyopathy or congestive heart failure such as ischemic heart disease.

### Diagnosis

Echocardiogram is useful to assess cardiac function and structure when diagnosing cardiomyopathy. However, cardiac MRI is now the gold standard for obtaining accurate cardiac dimensions and measuring function in most neuromuscular disorders because it is not hindered by anatomic abnormalities such as kyphosis [61].

### Treatment

In patients with suspected or documented heart failure, a referral to a specialist is appropriate. Heart failure medications improve cardiac parameters in neuromuscular patients with non-ischemic cardiomyopathy. The three most useful classes of drugs for reversing cardiac remodeling related to cardiomyopathy are angiotensin-converting enzyme (ACE) inhibitors or angiotensin-receptor blockers (ARBs), beta-adrenergic receptor blockers, and mineralocorticoid receptor antagonists (MRAs). Other guideline directed medical therapies for heart failure (i.e. digoxin, diuretics, and others) have also been shown to reduce morbidity and mortality in patients with non-ischemic cardiomyopathies with a reduced ejection fraction [61].

If cardiomyopathy progresses despite aggressive pharmacotherapies, other treatment considerations include implantation of an automated implantable cardioverter-defibrillator (AICD) with or without biventricular pacemaker, and implantable left ventricular assist devices (LVADs).

### Surveillance

Given the possibility that VCP patients with cardiomyopathy may be asymptomatic, it is reasonable to perform a cardiac MRI at baseline to determine present cardiac function. VCP patients should be monitored for symptoms of cardiomyopathy annually, and a repeat cardiac MRI can be performed if abnormal symptoms or clinical signs develop. For patients with detectable cardiac involvement on cardiac MRI such as delayed gadolinium enhancement in the ventricular wall or reduced ejection fraction, cardiac MRI should be repeated every 2 years to evaluate for cardiac remodeling following pharmacologic interventions.

## Respiratory dysfunction

### Clinical features

Respiratory muscle weakness occurs in at least 40–50% of patients with VCP MSP [64, 65]. Early manifestations of respiratory involvement may include recurrent tracheobronchitis and pneumonia due to weakness of the expiratory muscles with consequent impairment of cough strength and efficacy [66]. Progressive weakening of respiratory muscles leads to development of sleep disordered breathing characterized by nocturnal hypoventilation and oxygen desaturation [67]. In addition, patients with bulbar symptoms are at increased risk for aspiration. Ultimately, if not properly managed, these changes can result in respiratory failure and daytime alveolar hypoventilation (i.e. hypercapnia). Recognition and treatment of respiratory failure is critical since it is a frequent cause of death in VCP patients [24, 68].

### Diagnosis

Respiratory muscle function can be assessed using pulmonary function tests [PFTs, particularly forced vital capacity (FVC): sitting and supine], maximum inspiratory and expiratory muscle pressures (MIP and MEP), and peak cough flow (PCF). Sleep disordered breathing and nocturnal hypoventilation can be identified by either a full in-lab nocturnal polysomnography or limited at-home overnight monitoring. The presence of respiratory failure and hypercapnia is often assessed using arterial blood gas analysis, but this test may not be readily available in some outpatient settings. Alternatively, measurement of the serum bicarbonate in venous blood provides an easily obtainable and reliable marker of renal compensation for chronic carbon dioxide retention.

Bulbar and cognitive dysfunction increase risk for pulmonary complications, and limit ability to monitor patients with PFTs and sleep studies.

### Treatment

Extrapolating from management of respiratory dysfunction in other neuromuscular disorders, the treatment team should include a physical therapist, respiratory therapist, and a pulmonary specialist with expertise in management of patients with neuromuscular disease. The patient and their caregivers should maintain up to date vaccinations. Lung volume recruitment (LVR) and cough augmentation is important for the clearance of secretions and can be facilitated with LVR bag, mechanical insufflation/exsufflation (MI-E), and suction devices as needed. Respiratory infections should be treated early and aggressively. Non-invasive positive pressure ventilation (NIPPV), such as bilevel positive airway pressure (BiPAP) or volume assured pressure support (VAPS), should be initiated at the onset of nocturnal hypoventilation or with deterioration of respiratory function (e.g. arterial PCO_2_ $>45$  mmHg, FVC $<$ 50% of predicted, MIP $<$ − 60 cm H_2_O). Therapy with continuous positive airway pressure (CPAP) should be reserved for individuals that demonstrate obstructive sleep apnea in the absence of nocturnal hypoventilation. The role of respiratory muscle training to improve clinical outcomes is unclear and is currently being investigated in VCP MSP (curevcp.org).

Supplemental oxygen and/or therapy with respiratory depressants (e.g. sleep aids, narcotics, anxiolytics and antidepressants) should be withheld if possible as they lower central respiratory drive and can induce or worsen hypercapnia. Diuretic therapy may be used to reverse peripheral edema in patients with cor pulmonale. However, primary metabolic alkalosis with impaired central respiratory drive may ensue because of the associated chloride and potassium deficiency.

### Surveillance

Serum bicarbonate and PFTs can be checked annually in myopathy phenotype if stable, or every 3–6 months if deteriorating and/or ALS phenotype. A sleep study should be performed at baseline in patients with documented or symptomatic weakness.

## Supportive therapies (physical and occupational therapy, speech-language pathology, and respiratory therapy)

VCP MSP patients should have access to an individualized, comprehensive care plan with an interdisciplinary team including physical therapy (PT), occupational therapy (OT), speech language pathology (SLP), and respiratory therapy (RT). The impacts of VCP MSP across body structures and function should be routinely monitored and managed to optimize a patient’s independence with activities of daily living (ADLs) and quality of life (QOL). Clinical outcome assessments (COA) performed by trained practitioners are useful for assessing function and to track the level of independence and/or assistance required to complete ADLs. Use of standardized COAs also facilitate proactive planning for procurement of any needed assistive devices, home and work modifications, or other supports. Including a patient-reported outcome (PRO) measure with strong psychometric properties is also beneficial for assessing health indices and QOL and is recommended to be completed at least annually. Pain and fatigue PROs should also be monitored at least annually with quantitative severity scales as these may impact both physical and cognitive function. Standardized tools are preferred over generalized scales.

### Physical and occupational therapy

Physical and occupational therapy focus on helping patients maintain or adapt to changes in their function and ADLs to promote independence and QOL. While surveillance by PT and OT is recommended during multidisciplinary clinic visits, if there is difficulty with performing ADLs, mobility, or transfers, patients should be referred to PT and OT for ongoing treatment including appropriate exercises, home adaptations, and/or equipment. Regular and monitored aerobic exercise programs of low to moderate intensity are recommended to preserve function, strength, range of movement, endurance, balance, independence with ADLs, and participation [69, 70]. Inactivity can lead to further weakness and functional decline [71], so maintenance of current strength versus targeted muscle strengthening with proper monitoring may be indicated in some patients. While exercise is recommended there remains a lack of evidence for the most effective prescription in VCP MSP. Relevant assistive devices include ankle foot orthotics, canes, walkers, wheelchairs, chair seat risers, mobile arm supports, and hoists/ lift systems.

### Speech-language pathology

The assessment of bulbar function is crucial and closely tied to respiratory function and nutritional status. SLP measures include swallowing function, speaking rate, overall speech intelligibility. A swallow study (e.g. videofluoroscopy or flexible endoscopy) can identify difficulties with swallowing permitting targeted treatment. An augmentative and alternative communication (AAC) examination can be conducted to ensure optimal communication, as well as provide education and training for using various external communication tools, like an alphabet board, tablet, or speech-generating device.

### Respiratory therapy

RT is critical for monitoring and optimally managing respiratory function. PFTs should be performed at least annually, including measurements of FVC in sitting and supine positions. Chest physiotherapy, non-invasive ventilation, and insufflation–exsufflation devices should be initiated in a timely fashion.

## Mental health

VCP MSP patients are at risk for higher levels of stress, depression, and anxiety due to the inherent rare nature of the disease, which predisposes them to misdiagnosis, differing clinical opinions, lack of resources and information, economic burden, and isolation [72]. Compounding all of these issues is the absence of true disease modifying therapies for the major components of the disease, aside from bisphosphonates for PDB. However, appropriate therapeutic support can help patients manage and process their diagnosis, work towards short- and long-term goals to improve their quality of life, engage in peer or professional support groups, facilitate challenging conversations with family members, reduce the risk of self-harm, and validate feelings of fear, grief, and anger.

Evidence-based practice includes screening all patients for behavioral health signs and symptoms and referring to behavioral providers upon learning of their diagnosis [73]. Collaboration between the multidisciplinary team and community mental health providers ensures a holistic approach to supporting individuals living with VCP, both at the time of diagnosis and throughout their experience with the disease.

## Urinary and anal incontinence

VCP patients may experience urinary or anal incontinence/dysfunction as reported in isolated cases [26, 68], however is underrecognized. In other neuromuscular disorders, urinary and anal dysfunction are present and can significantly lower the quality of life for patients [74–76]. Screening for these symptoms during annual examinations through patient survey or interview may be appropriate and help to discern the etiology of the incontinence. Functional incontinence may require addressing issues of mobility, bathroom accessibility or scheduled voiding whereas other forms of incontinence may require referral to a specialist. Further studies are needed to determine the true prevalence of urinary and anal dysfunction in VCP patients.

## Supplements and nutrition

Specific diets and supplements have not been formally studied in VCP patients. A lipid enriched diet was shown to improve survival, motor activity, muscle pathology and the autophagy cascade in VCP^R155H/R155H^ homozygous mice [77], however this requires further study in humans. Given the oxidative stress that occurs in VCP MSP pathophysiology, it is reasonable to recommend an anti-inflammatory diet that is rich in fruits and vegetables, whole grains, lean proteins, and fatty fish, and avoid highly processed foods and products that contain preservatives, pesticides, and artificial ingredients. Supplements that may play a role in reducing oxidative stress include N-acetyl cysteine, alpha lipoic acid, acetyl-L-carnitine, vitamin B12, and coenzyme Q10, among others, although there remains a lack of evidence that these supplements alter symptoms or the course of the disease. In addition, there is evidence that a Mediterranean diet may offer protection in dementia and cardiovascular disease [78], which could be extrapolated with regards to VCP pathology, but further studies of this theory are needed. It is important to identify barriers to nutritional literacy and access to resources that may limit patients and caregivers understanding of dietary recommendations. A referral to a nutritionist or registered dietician may be appropriate.

## Conclusion

VCP MSP is a rare disease with diverse multi-organ phenotypes that warrants better awareness and management in the physician community. Our international multidisciplinary meeting in April 2021 established the standard of care recommendations for the various aspects of the disease from all relevant specialties.

Development of an optimal clinical outcome toolbox for assessment of patients with VCP MSP is necessary to better monitor and manage patients. It may be challenging to recruit a large sample size at any particular institution, but multisite clinical trials using a spoke and hub model with centralized administration and scoring of COAs could improve results. Similarly, utilizing COAs that can be validly administered and scored within a patient home environment can help reduce the burden of travel to distant sites for trial visits. Future large-scale studies are needed to better inform clinical practice guidelines on recommended treatments, therapies, and exercise programs, and to identify characteristics associated with responders to proposed interventions.

## Supplementary Information

**Additional file 1.** Named authors.

**Additional file 2.** Co-investigators and acknowledgements.

## Data Availability

Data sharing not applicable to this article as no datasets were generated or analyzed during the current study.
